# Investigation on the Indium–Tin Oxide Nanoparticle-Based Chemoresistive Sensors to Detect Small-Molecular-Weight Substances Diluted in Water

**DOI:** 10.3390/s26134066

**Published:** 2026-06-26

**Authors:** Yujin Song, Chanyoung Bae, Hyeonjun Lee, Mincheol Han, Moonjin Lee, Jae-Jin Park, Jiho Chang

**Affiliations:** 1 Major of Nano-Semiconductor Engineering, Korea Maritime and Ocean University, Busan 49112, Republic of Korea; yujin_song@g.kmou.ac.kr (Y.S.); chanyoung_bae@g.kmou.ac.kr (C.B.); hj30123@g.kmou.ac.kr (H.L.); mincheol_han@g.kmou.ac.kr (M.H.); 2 Maritime Safety and Environment Research Center, Korea Research Institute of Ships & Ocean Engineering (KRISO), Daejeon 34103, Republic of Korea; moonjin.lee@kriso.re.kr (M.L.); jaejinpark@kriso.re.kr (J.-J.P.)

**Keywords:** indium–tin oxide, small-molecular-weight substances, chemoresistive sensor

## Abstract

We fabricated a chemoresistive sensor based on indium–tin oxide (ITO) nanoparticle detection layer printed on a polyethylene terephthalate (PET). The ITO sensor operates on a mechanism that detects substances through resistance change induced by electrochemical potential variations on the sensor surface, which correspond to changes in analyte concentration governed by the Nernst equation. In this study, we confirmed broad-spectrum detection capabilities of the ITO sensor by successfully detecting 31 kinds of substances and demonstrated by achieving a low limit of detection that fully satisfies the environmental protection limit (EPL) for effluents, also alongside an error margin of within 5% for all 31 substances. In addition, the possibility of selective detection was confirmed by presenting the response of the ITO sensor according to pH changes, concentration, and type of substance as a two-dimensional scattering pattern. Thus, this study demonstrates that ITO based on chemoresistive sensors can achieve real-time monitoring of various underwater substances with high sensitivity and broad detection capabilities.

## 1. Introduction

Monitoring various noxious liquid-phase substances, such as industrial chemical compounds, petroleum, and pesticides, is essential for protecting human health and marine ecosystems [1]. While various sensors such as turbidity [2], dissolved oxygen [3], organic carbon [4], nitrogen/phosphorus [5,6], temperature [7], salinity [8], and pH [9] are developed for water quality monitoring and evaluation, it should be noted that there is still a lack of sensors capable of detecting small-molecular-weight substances (SMSs) in the field. The SMSs generally refer to relatively small molecules with a molecular weight of several hundred to 1000 Daltons (Da) or less, and include hydrocarbons, alcohols, solvents, inorganic compounds, pharmaceuticals, pesticides, and monomers [10]. This is because SMSs are extremely diverse, and since each compound varies in volatility, solubility, density, toxicity, and reactivity, it is virtually impossible to detect all compounds with a single sensor.

Table 1 summarizes the representative ITO-based sensing platforms reported in recent studies, including target analytes, detection ranges, LODs, and key features. Most previous studies have shown the performance of ITO-based sensors with high-sensitivity detection and low LOD of specific analytes. However, there is a lack of investigation into a wide range of organic low-molecular-weight substances and diluted metal ion solutions.

Conventional water quality sensors generally operate by detecting only specific signals in terms of pH, gas emissions, and spectral patterns. Consequently, these detectors may react to certain substances but not others, and detection sensitivity can be significantly reduced by variables such as salinity, water temperature, organic matter, suspended particles, and ocean currents.

To overcome these inherent limitations and achieve reliable SMS detection in complex water environments, developing a robust and versatile sensor is inevitable. In this study, we propose a chemoresistive sensor utilizing an indium–tin oxide (ITO) nanoparticle film fabricated on a polyethylene terephthalate (PET) substrate. This chemoresistive sensor offers the advantages of applying to portable, disposable, and wearable applications, and is particularly suitable for real-time detection [11].

Previous studies have demonstrated that ITO sensors can detect SMSs by monitoring their resistance changes (ΔR). It has been confirmed that organic solvents such as ethanol, methanol, and IPA diluted to concentrations of tens of ppm can be detected using an ITO sensor [12]. Additionally, ethanol diluted in seawater was also detected using ITO sensors, suggesting the potential applicability of ITO-based sensors in aqueous environmental conditions [13]. In this study, 31 types of SMSs and diluted metal ions representing dissolution (D), sink and dissolution (SD), and dissolution–evaporation (DE) behaviors were selected for the experiments.

**Table 1:** Comparison of ITO-based sensing platforms.

Sensor Platform	Target Analyte	Detection Range	LOD	Key Features	Ref.
Mn (III)-porphyrin-modified ITO electrode	Levofloxacin	10^−9^ M–10^−3^ M	4.82 × 10^−10^ M	High selectivity and excellent recovery	[14]
AuHgPt/ITO electrode	hydrogen peroxide	0.5 μM–5 mM	0.17 μM	High selectivity and excellent recovery	[15]
MgO nanoparticles/ITO electrodes	bisphenol A	50 nmol L^−1^–10 µmol L^−1^	1.13 nmol L^−1^	High selectivity and excellent recovery	[16]
CuNPs/NH_2_-VMSF/ITO	NO_3_^−^	5.0~1000 μM	2.3 μM	High selectivity and excellent recovery	[17]
ITO-based dual-mode (Optical/Electrochemical) biosensors	Carbonic Anhydrase IX	10 ng/mL–10 µg/mL	266.4 ng/mL	High selectivity and excellent recovery	[18]
ITO/Ag chemoresistive sensor	Organic small-molecular-weight substance and diluted metal ions	20 mg/L–160 mg/L	36.6 μg/L	Borad target, and simple structure	This work

In addition, to investigate whether it is possible to select specific SMS, we simultaneously measured sensor responses and pH for 10 types of SMSs. Using these results, we constructed a 2D map and verified whether using ITO sensor responses and pH together could provide additional information for distinguishing SMSs.

## 2. Materials and Methods

### 2.1. Fabrication of Chemoresistive Sensor

**Figure 1:**
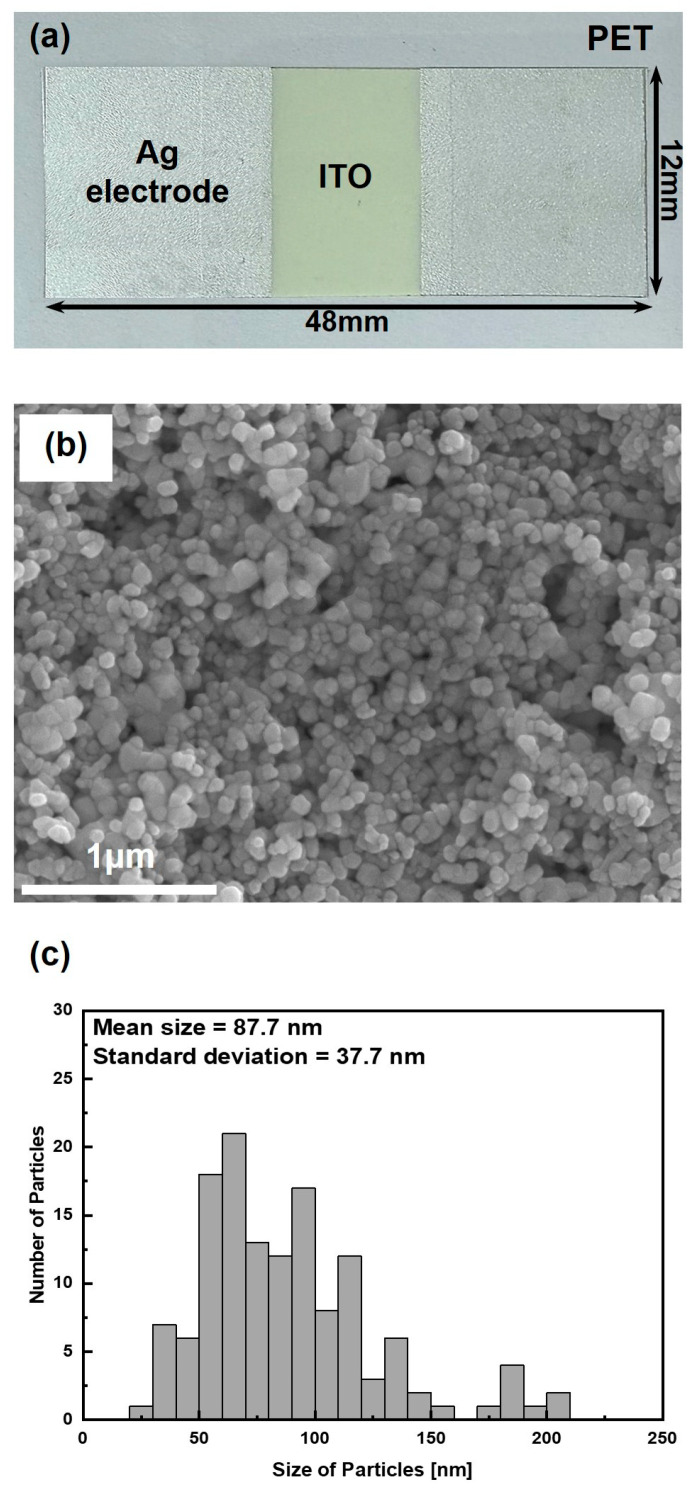
(**a**) Photograph of the ITO sensor, (**b**) SEM image of the ITO film surface, (**c**) histogram of particles size distribution.

The sensor was fabricated using a screen-printing technique on a polyethylene terephthalate (PET) film. The PET substrate was cleaned in deionized (DI) water for 15 min using an ultrasonic cleaner (SD-Ultrasonic Co., Ltd., Seoul, Republic of Korea). An organic binder was prepared by mixing α-terpineol (Kanto Chemical Co., Inc., Tokyo, Japan) and ethyl cellulose (Junsei Chemical Co., Ltd., Tokyo, Japan) in a weight ratio of 19:1, and a paste was prepared by mixing it with ITO nanoparticles in a weight ratio of 1:0.8. The ITO nanoparticles used products with the ratio of In_2_O_3_:SnO_2_ = 90:10 at 99.99% purity of US Research Nanomaterials, Inc.(Houston, TX, USA), and the particle size is 20–70 nm. The prepared ITO paste was printed onto the PET substrate (20 × 20 mm^2^) and heat-treated at 100 °C for 3 h to eliminate residual binders from the film’s surface. Also, the thickness of the manufactured ITO film was measured to be 12.25 μm. As shown in Figure 1a, silver (Ag) paste (JIN Chemical., Hwaseong, Republic of Korea) was printed (18 × 20 mm^2^) on both ends of the ITO film to enhance electrical contact and was cured at 80 °C for 1 h. To further remove surface contamination by residual binder, the film underwent an additional ultrasonic cleaning in DI water for 15 min. Previous studies have shown that removing residual organic binder improves particle packing density and reduces film resistivity, enhancing the sensor’s accuracy [19]. Figure 1b shows the surface topography of the ITO nanoparticle using SEM. Figure 1c shows a particle size distribution histogram used to analyze the grain size of the ITO nanoparticles. The mean diameter of the ITO nanoparticles was observed to be 87 nm with a standard deviation of 37.7 nm. It is well shown that nanoparticles are suitable for use as sensors because they have a larger contact area with liquid analytes than thin films.

### 2.2. Measurement Method of ITO Sensors

The Ag electrodes at both ends of the sensor were connected to an I-V meter (2400 Source Measurement Unit, Keithley Instruments, Inc., Solon, OH, USA). The specific electrical measurement conditions of the I-V meter were as follows: The voltage source function was used to apply a DC bias of 1 V in the 2-wire mode. The data were measured at an average interval of approximately 0.12 s, which corresponds to a sampling rate of approximately 8.3 Hz. In addition, the compliance current was set to 105 μA, the NPLC/speed to normal, and the measure delay to 0 s. The data were collected through a GPLB interface. The initial reference resistance (R_0_) was measured before applying any analyte and was approximately 50KΩ on average. A 35 μL droplet of an SMS-diluted analyte was applied to the sensor surface, and resistance change (ΔR) was monitored over time until saturation, which typically took about 600 s. Experiments analyzed the sensor response (ΔR) against varying SMS concentrations (20–160 ppm) to ensure measurements covered exposure tolerance limits for each substance. In preparing the aqueous solutions, each substance was diluted with DI water to concentrations of 20–160 ppm, and the physical properties (density and purity) of each substance were considered when calculating the required amount. In this study, 1 ppm of the dilute aqueous solution was considered the same as 1 mg/L. This concentration range was selected to examine the basic sensor response of the ITO sensor in the low-concentration region, as clear regulatory criteria for these substances in aqueous environments have not yet been fully established.

### 2.3. Operational Principles of the ITO Sensor

When an analyte contacts the ITO film surface, an electrochemical interaction occurs at the solid–liquid interface. The potential difference between the Fermi potential (E_f_) of the ITO film and the redox potential (E_redox_) of the analyte generates a built-in potential (V_bi_), as described in Equation (1):

V_bi_ = |E_f_ − E_redox_|/q (1)

This potential difference alters the depletion layer width, changing the surface carrier concentration (n_s_) and affecting the surface resistivity, as shown in Equation (2):

n_s_ = n_b_ exp(−qV_bi_/kT) (2)

where q is the unit charge, n_s_ is the ITO surface carrier concentration, n_b_ is the bulk carrier concentration, k is the Boltzmann constant, and T is the absolute temperature. The resistance change (ΔR) caused by these interactions can be expressed as

R_0_ = R_s_//R_B_ (3)

R = (R_s_ + ΔR_s_)//R_B_ (4)

ΔR = R/R_0_ = ((R_s_ + ΔR_s_)//R_B_)/(R_s_//R_B_) (5)

where R_0_ is the initial resistance, R_s_ is the surface resistance, R_B_ is the bulk resistance, ΔR_s_ is the surface resistance change by contacting the analyte, R is the measured resistance after contact the analyte, and ΔR represents the ratio of resistance change, which was defined as the sensor’s response [20].

In addition, the operating principle of the ITO sensor is based on the relationship between the redox potential of the analyte and the electrical state of the ITO sensing layer. In previous CV-based studies, the redox potentials of methanol, ethanol, and IPA were determined, and the values of the concentrations and the responses of the ITO sensor showed small errors of 1.4%, 3.5%, and 4.9%, respectively [21]. From these results, the response of the ITO sensor can be explained by the concentration-dependent change in the redox potential of the analyte, which can be interpreted as occurring due to the difference between the Fermi level of ITO and the internal potential between the analytes.

Additionally, pH measurements were conducted simultaneously using a commercial pH sensor. Each substance exhibits distinct pH behavior in aqueous solutions. We explored a method to improve the accuracy and selectivity of an ITO-based chemoresistive sensor for detecting SMSs in water by combining resistance change (ΔR) and pH measurement.

## 3. Results and Discussion

We investigated 31 types of organic low-molecular-weight substances and diluted metal ions representing dissolution (D), sink dissolution (SD), and dissolution–evaporation (DE) behaviors in water. D-Type (dissolving) means that they dissolve completely in water, DE-type (dissolving and evaporating) means that they evaporate while dissolving in water, and SD-type (sinking and dissolving) means that they precipitate while dissolving in water. Sensor responses of those substances were measured using ITO film sensors, respectively. Also, all diluted metal ions solutions were prepared by diluting commercially available standard solutions.

For evaluating sensor performance, eight performance parameters were included, such as sensor response (∆R), response time (τ), sensitivity (S = δ(∆R)/δ[C]), signal-to-noise ratio (S/N ratio), detection range, linearity (R^2^), limit of detection (LOD), and error (%). The response ∆R represents the ratio of the initial resistance (R_0_) to the resistance after analyte exposure at saturation (∆R = R/R_0_). Response time τ is defined as the time required for the resistance change to move from 10% to 90% of its saturation value. Sensitivity S represents the ratio of change in sensor response (δ(∆R)) to a change in concentration (δ[C]) and corresponds to the average value obtained in the study. The S/N ratio is calculated as 10log (∆R_Signal_/∆R_Noise_), indicating the strength of the signal (∆R_Signal_) relative to the noise (∆R_Noise_). In this study, an S/N ratio of 20 dB or higher is considered standard. The detection range is defined as the range in which a clear response is observed through the S/N ratio, and the saturation value reaches 90% or higher when using the ITO film sensor. Linearity R^2^ describes the correlation of the measured data and is determined by fitting a linear function. The minimum detection concentration is defined as the limit of detection (LOD = 3σ/m, σ is the standard deviation, m is the slope of the detection curve), following common criteria [22], to determine whether the ITO sensor is able to detect below the minimum allowable concentration standard. The error is calculated as the ratio of the measured value to the reference value, and in this study, a standard of within ±5% was adopted by referring to the standards of commercial sensors.

**Figure 2:**
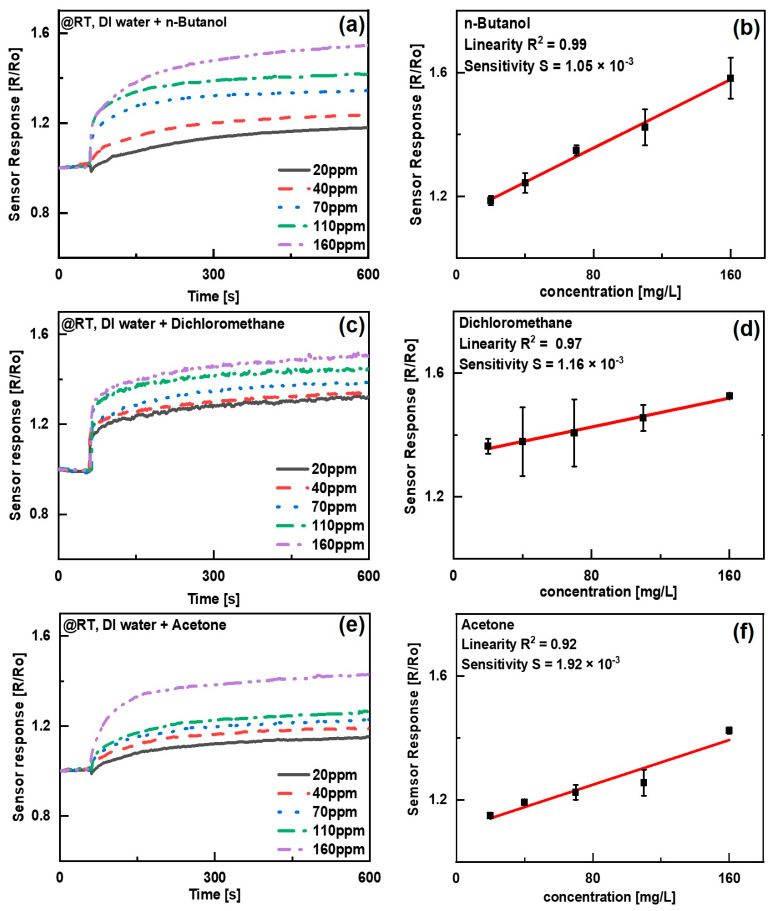
The sensor response for selected SMSs. Temporal change for (**a**) n-butanol, (**c**) dichloromethane, and (**e**) acetone, and corresponding sensor response for (**b**) n-butanol, (**d**) dichloromethane, and (**f**) acetone.

Figure 2 shows the sensor response of the ITO sensor according to the concentration change in three selected SMSs. Figure 2a shows the result of 1-butanol, a substance that completely dissolves in DI water (D-type). The sensor’s response remains stable for 60 s and then sharply increases resistance when in contact with the analyte (diluted 1-butanol). This resistance rise stabilizes and reaches saturation after approximately 500 s. Figure 2b presents a linear fitting (line) of the response change and measured responses (squares) according to the 1-butanol concentration. It shows strong linearity with R^2^ = 0.99, and the sensitivity S is confirmed to be 1.05 × 10^−3^. Figure 2c shows the measurement result of dichloromethane, a substance that dissolves and sinks in DI water (DS-type). Similarly to 1-butanol, when dichloromethane contacts the surface, the resistance sharply rises and then reaches saturation at around 700 s. Figure 2d presents a linear fitting (line) of the response change and experimental results (squares) along with dichloromethane concentration. It shows a linearity R^2^ = 0.97, and the sensitivity S is 1.16 × 10^−3^, respectively. Figure 2e shows the response of acetone, a substance that dissolves and evaporates in DI water (DE-type). The resistance rises sharply when it contacts the analyte and reaches saturation at around 460 s. Figure 2f presents a linear fitting (line) of the response change and experimental results (squares) according to the acetone concentration. It showed the lowest linearity (R^2^ = 0.92), but the sensitivity S was the highest at 1.92 × 10^−3^. While linear sensor characteristics were confirmed in all types of SMSs, it was found that the sensitivity and linearity of the sensor were affected by the underwater characteristics of the SMS.

Table 2 presents experimental results for 31 kinds of substances. As a reference, the background signal for DI water was determined as follows: The sensor response was 1.18 kΩ, the response time reached saturation at approximately 540 s, and the S/N ratio was 20.7 dB. In addition, a previous study [23] found that the accuracy of the sensor was about less than 2% on average, although it varied depending on the underwater behavior of the SMS.

In Table 2, the response ∆R and response time τ were measured at a concentration of 110 ppm for each SMSs. 1.4-dioxane had the lowest response (∆R = 1.26 KΩ), while cyanide had the highest response (∆R = 5.84 KΩ). The average sensor response (∆R) was 1.805 KΩ. Ethyl alcohol showed the longest τ of 910 s. Also, the lowest τ of 109 s was observed for manganese. The average τ was 333 s. Sensitivity *S* varied across substances. Ethyl alcohol exhibited the highest sensitivity (S = 3.87 × 10^−3^), while silver showed the lowest sensitivity (S = 0.5 × 10^−3^). The average sensitivity was (S = 4.51 × 10^−3^). Regarding the S/N ratio, all substances, except ammonia, chromium, bromodichloromethane, and mercury, met the standard value (>20 dB). The low signal-to-noise ratio of the SMS was temporarily attributed to external factors such as noise and humidity, as well as the small number of experiments. The detection range was 20 to 160 ppm for all substances. This result indicates that the sensitivity of the ITO sensor is significantly high for those 31 types of substances. The linearity R^2^ value was greater than 0.90 for all substances, except for trichloroethylene, ammonia, barium, and copper. Note that trichloroethylene and ammonia are highly volatile, which can cause concentration fluctuations and increase noise during the measurement. Also, barium and copper are SD-type substances. They would cause inaccuracy in the measurement, although further research is needed to explain in detail [24]. It was confirmed that the LOD values of all substances, excluding Hg, meet the environmental standards presented in this study. Table 2 summarizes the time-weighted average (TWA) exposure standards [25] and the permissible discharge limits set by South Korea’s Ministry of Environment [26] for the substances studied. The TWA value is calculated by multiplying the airborne measurement value of the hazardous factor by the occurrence time based on 8 h of work per day and dividing by 8 h. This standard refers to the “Standards for Classification, Labeling and Material Safety Data Sheets for Chemicals” organized based on the American Occupational Safety & Health Administration (OSHA) and the European Regulation on the Classification, Labeling and Packaging of Substances and Mixtures (EU CLP). It is important to note that TWA is a reference value for atmospheric conditions. Since regulatory values serving as standards for aqueous solutions of the substance under analysis are limited, the TWA value was temporarily used as a criterion to indicate the hazard of the substance. Although the number of experiments per substance was about five, which was not sufficient, it was confirmed that the error values of almost all substances were within ±5%. Therefore, these experimental results have shown that ITO sensors can detect environmental standard concentrations for various substances.

It is worth noting that the field application of this sensor can proceed as follows: First, continuous measurements for a targeted SMS are performed in the field using multiple ITO sensors. If the measured concentration of any substance exceeds minimum thresholds, a warning is activated to proceed with a more accurate analysis. Through this method, real-time monitoring of hazardous water substances in the field becomes possible, which was impossible with existing technologies.

However, water pollution spreads rapidly and contaminates a wide area fast [27]. Therefore, methods are needed to quickly identify pollution sources or narrow the target substance. To address this, this study investigated how to cross-reference the measurements of temperature, salinity, and pH, commonly used in water quality testing, with the ITO sensor measurements in this study to track contaminants. In this study, the pH change in the SMS analyte was measured, and the results were compared with those obtained from the ITO sensor.

**Figure 3:**
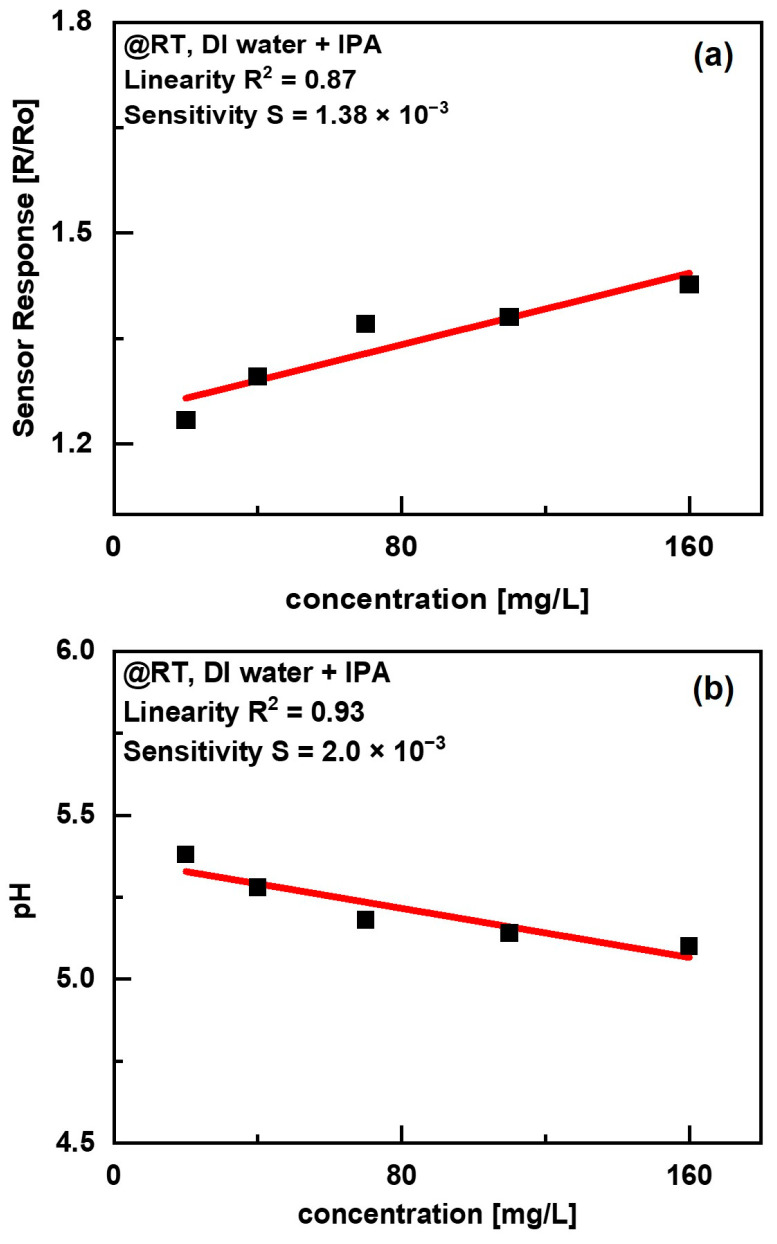
(**a**) Sensor response of IPA and (**b**) pH change depends on the concentration of IPA.

Figure 3 shows the ITO film sensor response and changes in pH value according to various IPA concentrations. IPA is fully soluble in water, and its permissible concentration based on the TWA standard is 200 ppm. The response change (Figure 3a) and pH change (Figure 3b) of the ITO film sensor are shown as the IPA concentration changes. The pH measurement value decreased as the IPA concentration increased. The R^2^ value is 0.93, showing a linear relationship with the IPA concentration change.

The strong correlation between pH and SMS concentration can be explained as follows: the Henderson–Hasselbalch Equation (6) below shows the relationship between pH and pK_a_ [28], where the pK_a_ is the acid dissociation constant.

(6) $$pH\;\;={pK}_{a}+log(\frac{\left[A^{-}\right]}{\left[HA\right]})$$

In Equation (6), we define [A^−^] as dissociated acid and [HA] as non-dissociated acid. This means that different solvents have varying concentration ratios of oxidizing and reducing species, leading to different E_redox_ differences, which can explain the observed variations in slopes. Therefore, it can be seen that the pH of the substance under analysis and the response value of the ITO sensor are correlated.

**Figure 4:**
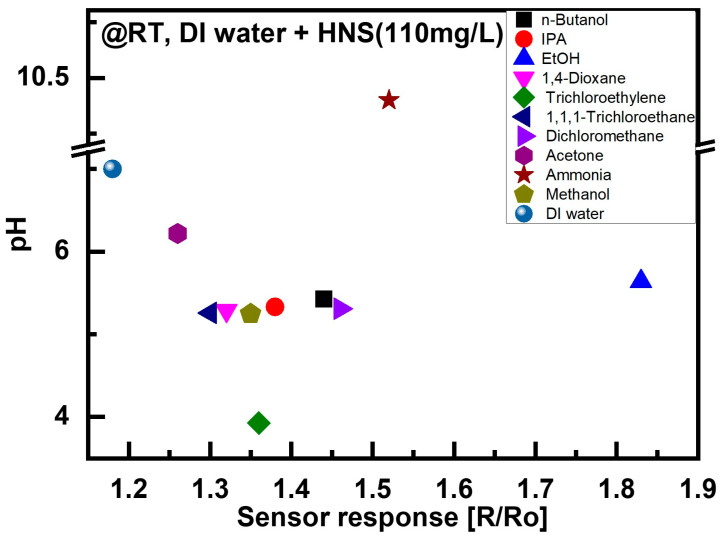
The two-dimensional scattering patterns of ITO sensor response and pH for 10 organic solvents and water.

Figure 4 shows the pH values according to the response values of the ITO film sensor of 10 selected SMSs. The pH value was measured when the concentration of each SMS was 110 mg/L The 2D mapping results suggest that combining the ITO sensor response with pH measurements or other measurements can be useful for distinguishing SMSs with similar sensor responses. For example, even for SMSs located within a relatively narrow pH range of 5.3 to 5.7, differences in ITO sensor responses can be utilized to differentiate these substances. Therefore, while these results demonstrate the potential to utilize multiple detection parameters for SMS differentiation, further statistical verification and improvements in detection capabilities are required for more reliable identification.

Table 3 summarizes the important parameters for 10 kinds of SMSs. The Standard European Behavior Classification System (SEBC) classifies SMSs based on their physical state, solubility in water, vapor pressure, and density [29]. Solvents can also be classified as polar or nonpolar. Solvents with a permittivity greater than 15 are considered polar, while solvents with a permittivity less than 15 are nonpolar. A higher permittivity indicates less interaction between oppositely charged ions in the solvent, and thus, permittivity is a measure of the solvent’s ability to insulate these ions. Polar solvents can be further divided into protic and aprotic. Protic solvents solvate anions by hydrogen bonding, while aprotic solvents solvate cations through negative dipoles. Therefore, the differences in permittivity between SMS types can induce pH changes, especially when combined with the response of the ITO sensor, which can help to distinguish specific SMSs. Therefore, it was found that by combining ITO sensors with conventional water quality assessment results, pollutants could be identified or limited to a few SMSs through field monitoring.

## 4. Conclusions

In this study, a chemoresistive sensor based on ITO film was developed and evaluated for the detection of 31 kinds of small-molecular-weight substances in water. ITO film sensors were shown to be capable of detecting concentrations below the permissible limit for various substances. Additionally, it was found that simultaneously measuring pH values provides additional information for determining SMSs. These results demonstrate that the ITO film sensor exhibits high sensitivity and wide detection capabilities, proving its potential for use in underwater SMS monitoring.

## Figures and Tables

**Table 2:** Summary of 31 kinds of substances and water with various underwater behaviors.

Substance	Response ∆R ^1^ (±SD) ^6^	Response Time τ [s] ^1^	Sensitivity S	S/N Ratio [dB]	Detection Range [ppm]	Linearity R ^2^	LOD [mg/L]	Error ^2^ [±%]	TWA ^3^ [ppm]	EPL ^4^ [mg/L]
1-Butanol	1.3101 (±0.05)	487	0.00105	21.8	20–160	0.99	0.0522	1.52	200	- ^5^
IPA	1.3803 (±0.05)	294	0.00138	27.6	20–160	0.93	0.0628	0.67	200	- ^5^
Ethyl alcohol	1.8867 (±0.03)	910	0.00387	32	20–160	0.95	0.0245	1.94	1000	- ^5^
1,4-Dioxane	1.2557 (±0.04)	535	0.00106	28.2	20–160	0.94	0.0416	1.88	20	4
Trichloroethylene	1.3043 (±0.02)	432	0.00094	21.1	20–160	0.89	0.056	0.71	10	0.3
1,1,1-Trichloroethane	1.3558 (±0.05)	429	0.00168	28.3	20–160	0.99	0.0096	4.48	350	0.1
Dichloromethane	1.4552 (±0.04)	747	0.00116	21.6	20–160	0.97	0.0175	0.63	50	0.2
Acetone	1.2662 (±0.04)	469	0.00196	23	20–160	0.92	0.048	3.81	500	- ^5^
Ammonia	1.52 (±0.03)	467	0.00262	18.1	20–160	0.76	0.0532	2.36	25	- ^5^
Methanol	1.3479 (±0.04)	482	0.00073	22.8	20–160	0.99	0.0118	1.28	200	- ^5^
Boron	1.29 (±0.04)	410	0.0018	30	20–160	0.95	0.0157	3.4	2	- ^5^
Chloroform	1.53 (±0.03)	568	0.0011	32	20–160	0.94	0.0459	2.4	5	0.8
Silver	1.3156 (±0.02)	136	0.0005	30	20–160	0.96	0.029	3.8	200	- ^5^
Phenol	2.05 (±0.09)	371	0.0066	27	20–160	0.91	0.0678	3.3	200	0.1
Methyl Ethyl Ketone	1.4402 (±0.06)	200	0.0013	21	20–160	0.95	0.052	4.6	10	- ^5^
Barium	1.776 (±0.06)	130	0.006	31	20–160	0.84	0.0693	1.1	- ^5^	1
Chromium	2.89 (±0.06)	145	0.0038	19.8	20–160	0.96	0.0165	3.4	- ^5^	0.2
Copper	1.837 (±0.03)	160	0.0009	28.1	20–160	0.86	0.0176	1.8	- ^5^	-^5^
Lead	2.504 (±0.05)	118	0.0018	29.5	20–160	0.86	0.0629	1.1	- ^5^	0.05
Manganese	1.678 (±0.05)	109	0.0027	22.5	20–160	0.93	0.0429	3.8	- ^5^	- ^5^
Zinc	1.412 (±0.01)	168	0.0012	25	20–160	0.95	0.0459	5.2	- ^5^	0.5
Tin	1.3 (±0.04)	131	0.0136	24	20–160	0.93	0.0399	2.5	- ^5^	0.5
Bromodichloromethane	1.907 (±0.04)	105	0.0061	19.7	20–160	0.97	0.0218	1.94	- ^5^	0.03
Dibromochloromethane	1.5913 (±0.06)	404	0.0027	35	20–160	0.92	0.0201	3.8	- ^5^	0.03
Cyanide	5.842 (±0.3)	600	0.0509	22	20–160	0.99	0.0076	5.1	- ^5^	0.1
Arsenic	3.012 (±0.13)	126	0.0046	25.5	20–160	0.9	0.0331	2.4	- ^5^	0.025
Cadmium	1.285 (±0.03)	145	0.0007	22.4	20–160	0.96	0.0157	3.4	- ^5^	0.01
Mercury	1.3385 (±0.1)	437	0.0026	18.8	20–160	0.99	0.0052	4.4	- ^5^	0.0005
Selenium	2.01 (±0.04)	140	0.0052	23.1	20–160	0.99	0.0118	2.1	- ^5^	0.1
Nickel	1.498 (±0.07)	165	0.0064	28	20–160	0.93	0.0338	3.7	- ^5^	0.3
Vanadium	2.37 (±0.03)	111	0.0029	27.7	20–160	0.94	0.0372	2.5	- ^5^	- ^5^
Water	1.18 (±0.03)	540	- ^5^	20.7	- ^5^	- ^5^	- ^5^	1.66	- ^5^	- ^5^

^1^ Measured at 110 ppm, ^2^ (true value—measured value)/true value × 100%, ^3^ TWA (time-weighted average), ^4^ EPL (environment permissible limits) for water discharge, Ministry of Environment Republic of Korea. ^5^ Not available. ^6^ Standard deviation (SD) based on 5 repeated measurements.

**Table 3:** Chemical properties of 10 kinds of SMSs including water.

SMS	Chemical Formula	Polarity Index	pKa	Dipole Moment [D]	Dielectric Constant	SEBC Type	pH ^1^
1-Butanol	C_4_H_10_O	3.9	16.10	1.66	17.1	D	5.43
IPA	C_3_H_8_O	3.9	16.5	1.66	15.7	D	5.33
Ethyl alcohol	C_2_H_6_O	5.2	16	1.69	24.55	D	5.64
1,4-Dioxane	C_4_H_8_O_2_	4.8	−3.9	0.45	2.209	D	5.29
Trichloroethylene	C_2_HCl_3_	1	- ^2^	- ^2^	3.4	SD	3.39
1,1,1-Trichloroethane	C_2_H_3_Cl_3_	- ^2^	- ^2^	- ^2^	7.5	SD	5.26
Dichloromethane	CH_2_Cl_2_	3.1	- ^2^	1.14	8.93	SD	5.31
Acetone	C_3_H_6_O	5.1	19.16	2.91	20.7	DE	6.22
Ammonia	NH_3_	- ^2^	36	1.47	16.9	DE	10.4
Methanol	CH_3_OH	5.1	15.5	1.69	32.70	DE	5.25
water	H_2_O	10.2	15.74	1.85	80.1	- ^2^	7

^1^ Measured at 110 ppm; ^2^ not available.

## Data Availability

The data presented in this study is available on request from the corresponding author. The data is not publicly available due to privacy restrictions.

## Abbreviations

ITO	Indium–Tin Oxide
PET	Polyethylene Terephthalate
EPL	The Environmental Protection Limit
SMS	Small-Molecular-Weight Substance
D	Dissolution
DE	Dissolution and Evaporation
SD	Sink and Dissolution
KIMST	The Korea Institute of Marine Science & Technology
